# Smokeless Tobacco Usage and Oral Cancer Risk: A Hospital-Based Case-Control Study From Bangladesh

**DOI:** 10.1177/1179173X251383752

**Published:** 2025-10-03

**Authors:** Md Zahid Ullah, Jennifer NW Lim, Marc Chrysanthou, Md Mostafizur Rahman, Md Saiful Arefin, Ashis Dhar, Md Sadat Hosen Shahriar

**Affiliations:** 1 Faculty of Education, Work and Health, 8695 University of Wolverhampton, Wolverhampton, UK; 2 Department of Oral & Maxillofacial Surgery, 469191 Shaheed Suhrawardy Medical College and Hospital, Dhaka, Bangladesh; 3 Department of Oral & Maxillofacial Surgery, 469193 Sher-E-Bangla Medical College, Barishal, Bangladesh; 4 Department of Neurological Surgery, University of Chicago, Maryland Ave, Chicago, USA; 5 Department of Health Education, 247328 NIPSOM, Dhaka, Bangladesh

**Keywords:** smokeless tobacco, chewing tobacco, betel quid, oral cancer, oral squamous cell carcinoma

## Abstract

**Objectives:**

Smokeless tobacco (SLT) use is common in South Asia and strongly associated with oral cancer. In Bangladesh, where SLT use is widespread, evidence remains limited. This study examined the association between SLT use and oral cancer among Bangladeshi adults and estimated the population-level burden.

**Methods:**

This first hospital-based case-control study from Bangladesh examined the association between SLT use and oral cancer. Conducted in Dhaka between July and December 2015. A total of 169 newly diagnosed oral cancer cases and 338 frequency-matched controls were recruited. Structured interviews collected data on SLT use, smoking, alcohol, BMI, and socio-demographic variables. Adjusted odds ratios (ORs) and 95% confidence intervals (CIs) were estimated using unconditional logistic regression. Population attributable fractions (PAFs) were calculated based on national SLT prevalence.

**Results:**

SLT use was strongly associated with oral cancer (adjusted OR: 8.78; 95% CI: 5.14-15.00). Risk was higher in women (OR: 14.33, 95% CI: 6.33-32.42) than in men (OR: 5.29, 95% CI: 2.62-10.67). Male dual users of SLT and smoked tobacco had the greatest risk (OR: 17.23, 95% CI: 5.70-52.01). Analysis by SLT type indicated significant independent associations with oral cancer for both Betel Quid (BQ) usage with tobacco (OR: 8.93, 95% CI: 5.23-15.27) and without tobacco (OR: 4.43, 95% CI: 1.94-10.10). A dose-response relationship was observed, particularly in women. SLT use accounted for an estimated 41% of male and 76% of female oral cancer cases in Bangladesh.

**Conclusion:**

SLT is a major, modifiable risk factor for oral cancer in Bangladesh, particularly among women and dual tobacco users. These findings support the need for stronger regulation, gender-sensitive education, and targeted SLT control strategies. Local evidence such as this is vital to shaping national and international oral cancer prevention policies.

## Introduction

Oral cancer ranks as the 11th most common cancer worldwide, accounting for an estimated 354 864 new cases and 177 394 deaths annually.^1^ The disease burden is disproportionately higher in developing countries, particularly in South Asia—home to Bangladesh, India, and Pakistan—where nearly two-thirds of cases occur.^2^ Bangladesh holds the third-highest incidence rate of oral cancer globally, with 9.5 cases per 100 000 population, and is the third leading cause of cancer-related mortality in the country. In 2018, approximately 9447 deaths were attributed to oral cancer in Bangladesh.^3^

Tobacco use, in both smoked and smokeless forms, is the single most important risk factor for oral cancer globally, accounting for the majority of cases.^4,5,6^ Of these, Smokeless tobacco (SLT) is of particular concern due to its widespread use, addictive nature, and substantial carcinogenic potential.^7,8,9,10^ Over 300 million people globally use SLT, with more than 90% of users residing in the WHO South-East Asia Region (SEAR).^11,12,13^ In Bangladesh alone, about 22 million adults consume SLT in various forms.^14^ SEAR consequently bears 85% of the global burden of SLT-related cancers, most notably in India, Bangladesh, and Pakistan.^11^ The health burden attributable to SLT in Bangladesh is, therefore, considerable.

SLT use in Bangladesh contributes significantly to disease burden, with over 16 including oral cancer and cardiovascular disease.^11^ Unlike smoking, the health effects of SLT vary substantially based on product type, composition, and processing methods.^11^ Although numerous international studies have confirmed SLT’s role in oral carcinogenesis,^4,15,16,17^ regional variations in product content complicate the extrapolation of global findings to Bangladesh.

Epidemiological studies from India and Pakistan have consistently shown a strong link between SLT and oral cancer,^8,10,18–21^ whereas research from countries like Sweden and the United States, where SLT products are chemically distinct, have reported weaker or non-significant associations.^22–25^ This discrepancy is largely attributed to the vast differences in carcinogenic content across SLT products, particularly the concentration of tobacco-specific nitrosamines (TSNAs), which can vary up to 400-fold.^26,27^ Additionally, the cancer-causing potential of SLT is influenced by processing techniques, including fermentation, curing, and temperature variations.^17^ Despite Bangladesh’s high SLT consumption and oral cancer rates, local studies investigating the SLT-oral cancer link remain limited. Recent analysis of 31 SLT brands in Bangladesh revealed TSNA concentrations higher than those found in comparable products from the United States and other South Asian nations.^27^ The need for context-specific data is therefore urgent.

To address this evidence gap, the present study reports the first hospital-based case–control investigation in Bangladesh quantifying the association between SLT use and oral cancer. It also estimates the population-attributable fraction of oral cancer cases attributable to SLT, providing essential evidence to inform national tobacco control policies and guide targeted interventions.

## Materials and Methods

### Study design and Setting

This hospital-based case-control study was conducted at a government dental college hospital in Dhaka, Bangladesh, between July and December 2015.

### Ethical Considerations

The study received ethical approval from the Anglia Ruskin University Research Ethics Committee and the participating hospital (Ref: NS/jc/FMSFREP/15-039). Written informed consent was obtained from all participants. For individuals who could not read, the consent form was read aloud in Bengali in the presence of a witness, and thumb impressions were used.

### Selection of Cases and Controls

A 1:2 case-to-control ratio was employed to maximise statistical power while minimising resource constraints. The required sample size was calculated using the Fleiss method for matched case–control studies. Based on prior survey data, a prevalence of SLT use among controls (p_0_) of 25% and among cases (p_1_) of 40% was assumed, corresponding to an odds ratio of approximately 2.0.^28^ With 90% power, a two-sided 95% confidence interval, and a 1:2 case–control ratio, the required sample size was 157 cases and 314 controls. Cases and controls were matched by age and gender to reduce confounding. In total, 169 histologically confirmed oral cancer cases and 338 matched controls were recruited, exceeding the minimum requirement. A post-hoc power calculation confirmed that the final sample provided >90% power to detect the observed effect size at α = 0.05.^29^

Inclusion criteria were adults aged 18 or older, born in Bangladesh, with no prior history of cancer. Exclusion criteria included individuals who were severely ill or unable to provide informed responses. Cases were newly diagnosed, biopsy-confirmed oral cancer patients classified according to the WHO ICD-10 system. These included cancers of the gums (C03), lower lip (C00.4), buccal mucosa (C06, left and right), tongue (C02 – lateral border; C02 – middle; C01 – base), floor of the mouth (C04), soft palate (C05), and overlapping regions of the oral cavity (C06).^30^

Controls were non-neoplastic patients attending the same hospital during the study period. This approach was chosen to ensure comparability of exposure recall and healthcare-seeking behaviour, and to reduce selection bias given the shared hospital catchment.^31^

### Data Collection

Interviewers were trained in standardised data collection procedures, and pilot testing was conducted to ensure consistency. The case participation rate was 92.86%, and the control participation rate exceeded 95%, minimising the risk of non-response bias. Interviewers conducted face-to-face interviews using a structured questionnaire adapted from the HeNce Lifestyle Study. Information was collected on socio-demographic variables, SLT use, smoking, alcohol consumption, body mass index (BMI), and family cancer history. A life grid approach was used to enhance recall accuracy. All data were anonymised and stored securely.^32,33^ The full questionnaire is available as Supplemental File 2.

### Variables and Definitions

The primary outcome variable was oral cancer status, confirmed through histopathological diagnosis. Cases were identified as individuals with malignancies in the lip, tongue, gingiva, floor of mouth, palate, or other parts of the oral cavity as classified by ICD-10 codes C00–C06.^34^ Gender-specific stratified analyses and interaction terms were also evaluated to explore effect modification by sex. Dual tobacco use (SLT and smoked tobacco) was defined as concurrent use of both forms for at least 1 year, and its association with oral cancer was assessed separately.

SLT was defined as non-combustible tobacco consumed orally or nasally. Betel nut, betel leaf, or pan masala without tobacco was excluded from SLT categories. Participants who used SLT for at least 1 year were classified as “ever users”; those who never used SLT or used it for less than 1 year were considered “non-users.” To quantify cumulative SLT exposure, the “chew-years” variable was calculated: Chew-years = (Daily frequency × Years of use)/10. This standardised exposure to a pack-equivalent metric.

Regarding smoking behaviour, an individual was categorised as an “ever smoker” of cigarettes/bidi if they had reported smoking for at least 1 year at any point in their lifetime.^35^ Although alcohol drinking is a well-known risk factor for oral cancer, this variable was excluded from the disease model due to the limited number of responses in this category. Only 1.5% of the total respondents, which is 8 people, reported ever drinking alcohol.

BMI was calculated from measured height and weight and categorised using WHO Asian cut-off values: <18.5 (underweight), 18.5-22.9 (normal), 23.0-27.4 (overweight), and ≥27.5 (obese). The full dataset used in this study, containing all variables included in the statistical analyses, is provided in the supplementary material (Supplemental File 1).

### Bias Control

To minimise selection bias, only biopsy-confirmed, newly diagnosed oral cancer cases were included. All incident cases presenting during the study period were invited to participate, reducing the risk of Neyman’s bias.^36^ Controls were recruited from the outpatient department of the same tertiary-level dental hospital to ensure comparability in healthcare-seeking behaviour and exposure recall. Although the use of hospital controls may raise concerns of Berkson’s bias, several steps were taken to minimise this risk. First, we excluded controls with pre-malignant lesions, oral cancer, or systemic diseases strongly linked to tobacco or alcohol. Second, controls were selected from a range of non-neoplastic dental conditions not causally related to SLT or smoking. Third, cases and controls were matched by age and gender to reduce confounding. Importantly, the prevalence of SLT use (26.3%) and smoking (15.4%) among controls closely mirrored national estimates (27.2% and 18.0%, respectively),^37^ suggesting good representativeness of the base population. While the possibility of Berkson’s bias cannot be entirely ruled out, these measures strengthen confidence that the observed associations reflect true population-level risks.^38^

To mitigate information bias, several strategies were employed. Recall bias was reduced by recruiting only newly diagnosed cases within 1 week of diagnosis and by interviewing controls during the same study window. A validated, structured questionnaire was administered face-to-face, incorporating a life-grid technique that anchored tobacco use history to major life events, thereby improving recall accuracy. Interviewer bias was minimised through training in neutral questioning and by blinding interviewers to the study’s exposure–outcome hypothesis. To further reduce the risk of differential probing, cases and controls were interviewed separately.^39^

Matching was performed on age and gender, and additional confounding variables such as education, BMI, employment, and smoking were adjusted for in multivariate models. A variable was considered a confounder if the adjusted odds ratio (OR) differed from the crude estimate by 15%-20% or more.^40^ High response rates reduced the likelihood of non-response bias.

### Statistical Analysis

All analyses were performed using SPSS version 24. Descriptive statistics were used to summarise the characteristics of cases and controls. Categorical variables were compared using chi-square or Fisher’s exact tests; continuous variables using t-tests.

Unconditional logistic regression was employed to estimate odds ratios (ORs) and 95% confidence intervals (CIs) for the association between SLT use and oral cancer. Multivariate models adjusted for potential confounders including age, gender, education, BMI, employment status, and smoking. Confounders were retained in the final model if they altered the crude OR by ≥ 15%. Unconditional regression analysis is regarded as the appropriate method when the age and gender distribution of the study subjects are not significantly different.^41^ Collinearity diagnostics were conducted for age of initiation, duration of use, and lifetime chew-years, with variance inflation factors (VIF) < 2.0 for all variables, indicating no multicollinearity concerns.

To explore potential effect modification, interaction between SLT use and smoking was assessed on both multiplicative and additive scales. Interaction terms were included in the logistic regression models, and stratified analyses by gender were also conducted to evaluate sex-specific associations. The interaction term that was tested was “SLT use status (dichotomous)*smoking (dichotomous)” and “cumulative exposure to SLT (dichotomous)* smoking (dichotomous)”.

The population attributable fraction (PAF) for Bangladeshi subjects was calculated using the Odds ratio derived from the unconditional logistic regression model and the prevalence of SLT from the National Tobacco Survey of Bangladesh.^34^ The following formula was used to calculate the PAF^31^:

$$PAF=\frac{\left[P\left(OR-1\right)\right]}{\left[P\left(OR-1\right)+1\right]}$$

Later, the total number of attributable incidence cases (AC) of oral cancer was calculated using the formula AC = PAF * TC. Here, TC is the total number of annual incident cases of oral cancer. The estimated annual incidence cases of oral cancer were obtained from the Global Cancer Observatory in 2018. A complete case analysis approach was used. No data imputation was performed, and analyses excluded records with missing key exposure or outcome variables.

## Results

### Participant Flow and Recruitment

During the study period (July–December 2015), 182 newly diagnosed oral cancer cases were identified. Of these, 13 patients declined participation due to time constraints or referral to other cancer hospitals, and 3 were unable to consent due to mental conditions. The final sample included 169 cases, yielding a case participation rate of 92.9%. The control group comprised 338 hospital patients admitted for conditions unrelated to SLT risk factors, mostly dental conditions under ICD-10 digestive system diseases (K00–K93; 88.8%), followed by injury/poisoning (S00–T98; 9.2%) and nervous system diseases (G00–G99; 2.1%)^30^. The full sample is presented in Table 1 (baseline characteristics). Of the 169 cases and 338 controls, 18 cases and 21 controls reported chewing betel quid without tobacco. As this exposure was not classified as SLT, these participants were excluded from the analyses presented in Tables 2 and 3. Therefore, the totals in those tables do not equal the overall sample size. Table 4 presents the joint effect of smoking and SLT among men only and therefore reflects a subset of the study population.

**Table 1.** Descriptive Characteristics of the Study Population

Characteristics	Cases (%)	Controls (%)	*P-*value
Gender			—
Male	81 (47.9)	162 (47.9)	
Female	88 (52.1)	176 (52.1)	
Age			—
Mean age^a^	53.97 ± 12.47	53.96 ± 12.55	
Education			**<0.001** ^b^
No-formal education	98 (58.0)	141 (41.7)	
Primary school completed or less	47 (27.9)	100 (29.6)	
High school completed	18 (10.6)	62 (18.3)	
More than high school completed	6 (3.5)	35 (10.4)	
Work status			0.27^b^
House worker	68 (40.2)	141 (41.7)	
Salaried worker	36 (21.3)	65 (19.2)	
Self-employed	28 (16.6)	50 (14.3)	
Farming	17 (10.1)	35 (10.4)	
Unemployed	20 (11.8)	47 (13.9)	
BMI (kg/m^2^)			**<0.001** ^b^
Normal weight (18.50 -24.99)	68 (48.9)	91 (72.8)	
Under-weight (<18.50)	52 (37.4)	10 (8.0)	
Over-weight (≥25)	19 (13.7)	24 (19.2)	
Cancer site (ICD-10)			—
Tongue (C01-C02)	5 (3.0)	—	
Floor of the mouth (C04)	2 (1.2)	—	
Gum (C03)	20 (11.8)	—	
Soft palate(C05)	5 (3.0)	—	
Lower lip (C00.04)	2 (1.2)	—	
Cancer overlapping region (C06)	135 (79.8)	—	
Grade of cancer			—
Grade I	103 (61.0)	—	
Grade II	58 (34.3)	—	
Grade III	8 (4.7)	—	
Smoking status			**<0.001** ^b^
Never	122 (72.2)	286 (84.6)	
Ever	47 (27.8)	52 (15.4)	
Alcohol drinking habit			0.21^b^
Never	164 (97.0)	335 (99.1)	
Ever	5 (3.0)	3 (0.9)	
Family history of cancer			0.53^b^
None	166 (98.2)	329 (97.3)	
Yes	3 (1.8)	9 (2.7)	

Abbreviation: ICD-10 International Classification of Diseases 10th Revision, BMI- Body Mass Index.

^a^Mean with Standard deviation.

^b^p-value derived from chi-square test/Fisher-exact test.

**Table 2.** Distribution of Smokeless Tobacco Use by Gender Among Cases and Controls

Categories	Males			Females			Total		
	Cases (%)	Controls (%)	*P*-value	Cases (%)	Controls (%)	*P*-value	Cases (%)	Controls (%)	*P*-value
Chewing habit with SLT^a^									
Never	**20 (27.4)**	**103 (67.3)**	**<0.001** ^c^	**10 (12.8)**	**110 (67.1)**	**<0.001** ^c^	**30 (19.9)**	**213 (67.2)**	**<0.001** ^c^
Ever chewers	53 (72.6)	50 (32.7)		68 (87.2)	54 (32.9)		121 (80.1)	104 (32.8)	
Type of chewing products^b^									
BQ with zarda	48 (59.3)	46 (28.4)	**<0.001** ^c^	57 (64.8)	50 (28.4)	**<0.001** ^c^	105 (62.1)	96 (28.4)	**<0.001** ^c^
BQ without zarda	8 (9.9)	9 (5.6)		10 (11.4)	12 (6.8)		18 (10.7)	21 (6.2)	
BQ with sadapata	3 (3.7)	2 (1.2)		8 (9.0)	1 (0.6)		11 (6.5)	3 (0.9)	
Gul/Panmasala	2 (2.5)	2 (1.2)		3 (3.4)	3 (1.7)		5 (3.0)	5 (1.5)	
Age at SLT initiation^a^									
Mean age	24.30 ± 8.56	24.36 ± 10.49	0.98^d^	21.59 ± 6.52	22.43 ± 8.50	0.54^d^	22.78 ± 7.57	23.35 ± 9.5	0.61^d^
≤20 years old	28 (38.4)	26 (17.0)	**<0.001** ^c^	46 (59.0)	32 (19.5)	**<0.001** ^c^	74 (49.0)	58 (18.3)	**<0.001** ^c^
21-30 years old	16 (21.9)	17 (11.1)		15 (19.2)	13 (7.9)		31 (20.5)	30 (9.5)	
>30 years old	9 (12.3)	7 (4.6)		7 (9.0)	9 (5.5)		16 (10.6)	16 (5.0)	
Freq of SLT use^a^									
Average per day	8.08 ± 4.17	6.60 ± 4.62	0.09^d^	9.32 ± 5.19	6.09 ± 5.50	**0.001** ^d^	8.78 ± 4.79	6.33 ± 5.09	**<0.001** ^d^
1-5 per day	19 (26.0)	29 (19.0)	**<0.001** ^c^	16 (20.5)	36 (22.0)	**<0.001** ^c^	35 (23.2)	65 (20.5)	**<0.001** ^c^
>5 per day	34 (46.6)	21 (13.7)		52 (66.7)	18 (11.0)		86 (57.0)	39(12.3)	
SLT holding duration^a^									
Avg min of holding	14.17 ± 17.73	10.12 ± 7.20	0.14^d^	13.82 ± 9.50	10.46 ± 8.86	**0.04** ^d^	13.98 ± 13.67	10.30 ± 8.07	**0.01** ^d^
≤10 minutes	37 (50.7)	43 (28.1)	**<0.001** ^c^	44 (56.4)	44 (26.8)	**<0.001** ^c^	81 (53.6)	87 (27.4)	**<0.001** ^c^
>10 minutes	16 (21.9)	7 (4.6)		24 (30.8)	10 (6.1)		40 (26.5)	17 (5.4)	
Total yrs. of SLT use^a^									
Avg years of use	29.75 ± 13.45	31.46 ± 15.41	0.55^d^	30.82 ± 12.78	31.24 ± 14.37	0.41^d^	30.35 ± 13.08	31.34 ± 14.84	0.59^d^
≤30 years	27 (37.0)	26 (17.0)	**<0.001** ^c^	34 (43.6)	22 (13.4)	**<0.001** ^c^	61 (40.4)	48 (15.1)	**<0.001** ^c^
>30 years	26 (35.6)	24 (15.7)		34 (43.6)	32 (19.5)		60 (39.7)	56 (17.7)	
Lifetime exposure to SLT^a^									
Average Chew-years^a^	25.01 ± 20.36	21.62 ± 23.38	0.43^d^	30.53 ± 22.87	20.26 ± 22.19	**0.01** ^d^	28.11 ± 21.89	20.92 ± 22.67	**0.02** ^d^
≤20 chew-years	28 (38.4)	30 (19.6)	**<0.001** ^c^	26 (33.8)	35 (21.3)	**<0.001** ^c^	54 (36.0)	65 (20.5)	**<0.001** ^c^
21-40 chew-years	15 (20.5)	15 (9.8)		23 (28.6)	13 (7.9)		38 (24.7)	28 (8.8)	
>40 chew-years	10 (13.7)	5 (3.3)		19 (24.7)	6 (3.7)		29 (19.3)	11 (3.5)	

Abbreviations: BQ (Betel Quid); SLT, Smokeless Tobacco.

^a^Totals do not equal the overall sample size (169 cases, 338 controls) because participants who chewed betel quid without tobacco (18 cases, 21 controls) were excluded from this analysis.

^b^Analysis including both chewing habit with and without tobacco.

^c^p-value derived from chi-square test/Fisher-exact test.

^d^*P*-value derived from t-test.

**Table 3.** Odds Ratios With 95% Confidence Interval for Oral Cancer From Smokeless Tobacco Use

Categories	Males			Females			Overall		
	Cases	Controls	Adjusted OR (95%CI)^a^	Cases	Controls	Adjusted OR (95%CI)^a^	Cases	Controls	Adjusted OR (95%CI)^a^
Never chewed^b^	**20**	**103**	**1**	**10**	**110**	**1**	**30**	**213**	**1**
Chewing habits^c^									
Ever chewing	53	50	5.29 (2.62-10.67)	68	54	14.33 (6.33-32.42)	121	104	8.78 (5.14-15.0)
Type of SLT^d^									
BQ with zarda/sadapata	48	46	5.89 (2.87-12.10)	65	51	14.03 (6.10-32.25)	116	99	8.93 (5.23-15.27)
BQ without tobacco	8	9	2.78 (0.81-9.51)	10	12	8.58 (2.66-27.74)	18	21	4.43 (1.94-10.10)
Gul/Panmasala	2	2	2.92 (0.21-40.91)	3	3	10.89 (1.63-72.24)	5	5	6.39 (1.39-29.35)
Age at SLT initiation^c^									
≤20 years old	28	26	6.12 (2.67-13.95)	46	32	16.65 (6.83-40.55)	74	58	9.84 (5.47-17.72)
21-30 years old	16	17	4.61 (1.78-11.88)	15	13	11.63 (4.06-33.34)	31	30	6.98 (3.48-13.98)
>30 years old	9	7	4.50 (1.36-14.85)	7	9	11.70 (3.22-42.47)	16	16	7.31 (3.04-17.55)
*P-for trend* ^e^			*0.51*			0.92			0.48
Freq of SLT per day^c^									
1-5 per day	19	29	3.12 (1.34-7.21)	16	36	5.37 (2.07-13.89)	35	65	3.96 (2.13-7.37)
>5 per day	34	21	8.55 (3.75-19.48)	52	18	32.65 (12.84-83.02)	86	39	16.70 (9.08-30.72)
*P-for trend* ^e^			0.10			***0.003***			***0.001***
SLT holding duration^c^									
≤10 minutes	37	43	4.83 (2.30-10.11)	44	44	11.74 (5.06-27.24)	81	87	7.21 (4.18-12.45)
>10 minutes	16	7	7.31 (2.38-22.61)	24	10	29.53 (9.84-88.56)	40	17	15.18 (7.05-32.68)
*P-for trend* ^e^			0.31			0.06			***0.04***
Total yrs. of SLT use^c^									
1-30 years	27	26	4.85 (2.16-10.89)	34	22	16.32 (6.61-40.27)	61	48	8.81 (4.87-15.94)
>30 years	26	24	5.93 (2.45-14.35)	34	32	12.04 (4.62-31.32)	60	56	8.12 (4.29-15.38)
*P-for trend* ^e^			0.81			0.75			0.73
Lifetime exposure to SLT^c^									
≤20 chew-years	28	30	4.34 (1.98-9.51)	26	35	8.96 (3.65-22.01)	54	65	6.01 (3.36-10.76)
21-40 chew-years	15	15	5.65 (2.11-15.09)	23	13	20.91 (7.24-60.39)	38	28	10.58 (5.18-21.58)
>40 chew-years	10	5	13.34 (3.41-52.15)	19	6	49.98 (14.06-177.63)	29	11	26.84 (10.81-66.62)
*P-for trend* ^e^			0.19			***0.009***			***0.006***

Abbreviations: CI: confidence interval; N: total number; OR: odds ratio; SLT: Smokeless Tobacco; BQ: Betel Quid.

^a^OR and 95% CI were calculated using unconditional logistic regression, adjusted for age, gender, education status (cat), employment status (cat), Smoking status (cat), BMI (cat).

^b^Reference category.

^c^Totals do not equal the overall sample size (169 cases, 338 controls) because participants who chewed betel quid without tobacco (18 cases, 21 controls) were excluded from this analysis.

^d^Analysis including both chewing habit with and without tobacco.

^e^*P*-values for the linear trend was derived from including the variable as a continuous variable in the unconditional regression model.

**Table 4.** Joint Effects of Smoking and Smokeless Tobacco Use Among Male Participants

Ever smoked	Ever SLT	Cases (*n* = 81)	Controls (*n* = 162)	Adjusted ORs (95%CI)^a^
No	No	06	72	1.0
Yes	No	14	31	4.18 (1.42-12.26)*
No	Yes	28	38	6.78 (2.47-18.67)**
Yes	Yes	33	21	17.23 (5.70-52.01)**

n: total number; ORs: odds ratio; CI: confidence Interval; SLT: smokeless tobacco.

^a^ORs adjusted for age, education status, employment status, and BMI.

*P-Value less than 0.05, **P-value less than 0.001.

### Descriptive Characteristics

The distribution of socio-demographic variables between cases and controls is summarised in Table 1. The mean age of participants was 52.6 years (SD 11.8) for cases and 51.4 years (SD 12.0) for controls. Most participants were female (52.1%), with women comprising a higher proportion of cases than men. Cases were more likely to be less educated, unemployed, and have a lower BMI compared to controls.

Behavioural patterns of SLT use are summarised in Table 2. Among male cases, 72.6% reported ever using SLT compared to 32.7% of male controls, and among female cases, 87.2% reported use compared to 32.9% of female controls (*P* < 0.001 for both). Betel quid (BQ) with zarda was the most common SLT product among all users (62.1%), while BQ with sadapata was predominantly used by female cases. The mean age of SLT initiation was 24.3 years for males and 21.6 years for females, with 49% starting before the age of 20 (*P* < 0.001). The mean frequency of use was 8.08 times/day for males and 9.32 times/day for females, with 57% of cases using more than 5 times daily. A higher proportion of cases held SLT in the mouth for more than 10 minutes (26.5%) compared to controls (5.4%), and mean chew-years were significantly higher among cases (28.11 vs 20.92; *P* = 0.02).

### Primary Association

Table 3 shows the association between SLT use and the risk of oral cancer. Ever use of SLT was significantly associated with oral cancer (adjusted OR: 8.78; 95% CI: 5.14-15.0). The crude OR was (OR: 7.96; 95% CI: 5.02-12.40) and adjustment for education, BMI, smoking, and employment status increased the strength of the association. When stratified by gender, the association was stronger in women (adjusted OR: 14.33; 95% CI: 6.33-32.42) than in men (adjusted OR: 5.29; 95% CI: 2.62-10.67), indicating potential sex-specific vulnerability.

### Product-specific and Dual Use

The associations were further examined based on SLT product types and concurrent smoking. Both SLT products with tobacco (adjusted OR: 8.93; 95% CI: 5.23-15.27) and without tobacco (adjusted OR: 4.43; 95% CI: 1.94-10.10) were independently associated with oral cancer. BQ combined with zarda or sadapata exhibited the highest risk levels. Among men, dual users of SLT and smoked tobacco had the highest observed odds of oral cancer, consistent with a markedly increased risk compared with either exposure alone. Table 4 shows that both smoking and SLT use independently increased risk, but their combined use resulted in a substantially higher adjusted odds ratio (OR = 17.23; 95% CI: 5.70-52.01). This possible synergistic interaction remained significant (p for interaction <0.001) after adjusting for confounders, underscoring the importance of targeting dual tobacco users in prevention efforts.

### Dose-Response Relationship

We assessed whether oral cancer risk increased with greater SLT exposure. A clear trend of increasing odds of oral cancer was observed with higher cumulative SLT exposure, as measured by chew-years. Individuals in the highest quartile had the highest odds, particularly among women, suggesting a dose-dependent association, although the case-control design limits causal inference.

### Population Attributable Risk

To estimate the potential public health impact, we calculated population attributable fractions based on national prevalence and risk estimates, while acknowledging that PAFs assume a causal relationship and should be interpreted cautiously in the context of a case-control study. The overall population attributable fraction (PAF) for SLT use and oral cancer in Bangladesh was 61%. Gender-specific PAFs were 41% for men and 76% for women. Among men, 72% of oral cancer cases were attributable to dual use of SLT and smoking. Based on 2018 national incidence estimates (13 401 new oral cancer cases), approximately 8174 cases were attributable to SLT use. This comprised approximately 3,646 cases in men and 3,424 cases in women attributable to SLT use. Among men, dual use of SLT and smoking was associated with around 6,378 cases. Because dual users are included within the overall SLT-attributable group, this figure is not additive but highlights the substantial contribution of dual use to the male burden.

## Discussion

### Key Findings and Interpretation

This study provides compelling evidence that SLT use is a significant independent risk factor for oral cancer in Bangladesh, supporting the IARC’s conclusion that SLT causes oral cancer in humans.^42^ The association remained significant after adjusting for demographic and behavioural confounders, and was consistently observed across subgroups, reinforcing findings from previous studies in India and Pakistan.^10,20,43^

A notable finding is the markedly stronger association between SLT use and oral cancer in women (OR: 14.33; 95% CI: 6.33-32.42) than in men (OR: 5.29; 95% CI: 2.62-10.67), echoing patterns seen across South Asia.^44,45^ Balaram et al. reported a stronger association of SLT with oral cancer in women (OR: 42; 95% CI: 24-76) than in men (OR: 5.1; 95% CI: 3.4-7.8).^45^ Similarly, Sinha et al. found higher odds for female SLT users (OR: 5.83; 95% CI: 2.93-11.58) compared to males (OR: 4.44; 95% CI: 3.51-5.61). The higher odds among women are likely influenced by distinct SLT consumption patterns. Notably, women started using SLT at an earlier age and consumed it more frequently than men. On average, female cases in this study initiated SLT use 2 years earlier than male cases. Additionally, 66% of women used SLT more than 5 times per day, compared to only 46% of men.

The gender-specific vulnerability observed in this study may also reflect deeper sociocultural dynamics. In many rural Bangladeshi communities, SLT is viewed not as a harmful substance but as a remedy for common ailments such as morning sickness, toothache, or anxiety.^46,47,48^ These misconceptions, particularly prevalent among women and those with limited formal education, contribute to early initiation and high-frequency use. Such patterns highlight a pressing need for health promotion strategies that go beyond regulation, incorporating culturally tailored education campaigns that challenge prevailing beliefs and raise awareness about SLT-related health risks. Community-based interventions, particularly those engaging women’s groups and informal health workers, could be especially impactful.

Both chewing BQ, with or without tobacco, were shown to be an independent risk factor for oral cancer, as identified in previous studies.^19,49,50^ This suggests that while tobacco-specific nitrosamines (TSNAs) substantially elevate carcinogenicity, the areca nut itself, widely used in all BQ preparations, is also a major contributor to oral cancer risk. Areca nut, the seed of Areca catechu, is classified by IARC as a Group I carcinogen^16,19,49,50,51,52^ and its carcinogenicity is independent of added tobacco. Importantly, the observed variation in risk across SLT product types has direct implications for public education and regulation. Products perceived as “natural” or “tobacco-free” may still carry significant cancer risk, which is often underestimated by users. This highlights the need for clear risk communication and consistent regulation across all SLT formulations, regardless of tobacco content.

A consistent dose-response relationship, similar to prior studies, was observed for both the intensity and duration of SLT use, further supporting the strength of the association. This mirrors findings from other South Asian studies, where prolonged or intense SLT use significantly increased oral cancer risk.^19,45,53^ Evidence from a case-control study in Pakistan showed that over 20 pack-years of Naswar use was associated with a 28-fold increase in the odds of oral cancer (95% CI: 9.3-90.2).^10^

A relatively lower odds ratio was observed in an Indian study, where SLT use for 40 years or more was associated with an eleven-fold increase in the odds of oral cancer.^53^ These variations likely reflect differences in SLT product composition across countries. In our study, women who used SLT more than 5 times per day had significantly higher odds of developing oral cancer (OR: 32.00; 95% CI: 12.84-83.02), which may suggesting greater biological susceptibility to oral tissue damage among women andmeriting further investigation.^45^

Overall, the PAF estimated in this study (61%) is higher than the pooled estimate of 45% reported in a recent South Asian meta-analysis, underscoring the particularly heavy burden of SLT in Bangladesh.^54^ Current study estimates that SLT use accounts for approximately 76% of oral cancer cases in Bangladeshi women and 41% in men. Additionally, 72% of oral cancer cases in men were associated with the dual use of SLT and smoked tobacco. These findings highlight the significant contribution of SLT use among women and dual tobacco use among men to Bangladesh’s oral cancer burden. A comparison with Pakistan reveals a contrasting gender-attributable risk pattern, where men have a higher attributable risk (68%) compared to women (38%). This discrepancy may be linked to the predominant use of naswar among Pakistani men, which differs in chemical composition from SLT products commonly consumed in Bangladesh.^10^

Our findings also highlight critical gaps in national research and surveillance systems. While SLT is widely consumed in Bangladesh, routine surveillance of SLT-related cancers remains limited, and national surveys often lack detail on product types, frequency, and duration of use. Additionally, there is little routine monitoring of the chemical composition and carcinogen load of SLT products, despite evidence of high TSNA concentrations in locally available brands.^27^ To develop effective, evidence-based policies, Bangladesh would benefit from establishing prospective cohorts, strengthening oral cancer registries, and investing in laboratory infrastructure to track the toxicity of SLT products. These steps would enable more precise risk stratification and better-targeted prevention strategies.

### Strengths and Limitations

This study has several limitations that should be acknowledged. First, as a hospital-based case–control study, the oral cancer cases may not fully represent the base population. Given that the study hospital is a tertiary-level dental facility catering primarily to patients with dental issues, the potential for selection bias (including Berkson’s bias) cannot be fully ruled out. The direction of this bias is difficult to determine, and the estimated associations may therefore be either overestimated or underestimated.^55^ However, the prevalence of SLT and smoking among controls closely mirrored national estimates, suggesting reasonable representativeness of the base population. Recruiting population-based controls would have required substantially greater resources and was not feasible within this study design.^56^

Second, recall bias is possible because exposure data were self-reported, a common limitation in case–control studies.^57^ We attempted to minimise this by including only newly diagnosed cases and by using a life-grid technique, which improves recall by anchoring exposure history to significant life events. Third, although multivariable adjustments were made, residual confounding may remain, particularly for unmeasured variables such as diet, oral hygiene, and occupational exposures.^40^ A further limitation is that some risk estimates had wide confidence intervals, reflecting limited precision. Finally, as with other observational case–control studies, causal inference cannot be firmly established.^28^

Despite these limitations, the study also has notable strengths. These include histologically confirmed oral cancer cases, a relatively large sample size, frequency-matched controls, high participation rates, and detailed exposure measurement. The dose–response relationships observed, along with consistent findings with regional studies, further strengthen confidence in the validity of the results.

## Conclusion

This study provides the first hospital-based case–control evidence from Bangladesh demonstrating that smokeless tobacco (SLT) use is a major driver of oral cancer. Strong dose–response effects were observed, with particularly high risks among dual users of SLT and smoking. More than 60% of oral cancer cases in this population were attributable to SLT use, underscoring its substantial contribution to the national cancer burden. Eliminating SLT use among women alone could potentially prevent three-quarters of oral cancer cases in this population. These findings highlight the urgent need for tobacco control measures in Bangladesh that address both smoked and smokeless forms of tobacco. A coordinated, gender-responsive national strategy is required, incorporating stricter regulation of SLT advertising and sales, higher taxation, stronger packaging and warning labels, and widespread public health education. Without such measures, SLT use will continue to drive preventable cancer cases and impose an enormous economic burden.

## Supplemental Material

Supplemental Material - Smokeless Tobacco Usage and Oral Cancer Risk: A Hospital-Based Case-Control Study From Bangladesh Supplemental Material for Smokeless Tobacco Usage and Oral Cancer Risk: A Hospital-Based Case-Control Study From Bangladesh by Md Zahid Ullah, Jennifer NW Lim, Marc Chrysanthou, Md Mostafizur Rahman, Md Saiful Arefin, Ashis Dhar, Md Sadat Hosen Shahriar in Tobacco Use Insights

Supplemental Material - Smokeless Tobacco Usage and Oral Cancer Risk: A Hospital-Based Case-Control Study From Bangladesh Supplemental Material for Smokeless Tobacco Usage and Oral Cancer Risk: A Hospital-Based Case-Control Study From Bangladesh by Md Zahid Ullah, Jennifer NW Lim, Marc Chrysanthou, Md Mostafizur Rahman, Md Saiful Arefin, Ashis Dhar, Md Sadat Hosen Shahriar in Tobacco Use Insights

## Data Availability

The anonymised dataset supporting the conclusions of this study is provided as supplementary material (Supplemental File 1). The dataset includes variables relevant to participant demographics, exposure status, and outcomes and is provided in Microsoft Excel (.xlsx) format.
