# Supplementary material for: Smokeless Tobacco Usage and Oral Cancer Risk: A Hospital-Based Case-Control Study From Bangladesh
Source: Tob Use Insights. 2025 Oct 3;18:1179173X251383752. doi: 10.1177/1179173X251383752 (PMC12495209; doi:10.1177/1179173X251383752)
Supplement: Supplemental Material - Smokeless Tobacco Usage and Oral Cancer Risk: A Hospital-Based Case-Control Study From Bangladesh [file sj-pdf-2-tui-10.1177_1179173X251383752.pdf]

Section A. Medical information

Interviewer Reminder: Prior to interview, obtain the information below from hospital medical records

Identification number: .... [ ][ ][ ][ ]

A1 Status.. [ ][ ]

(01) Case (02) Control

FOR CONTROL:

A2. Control Department: (*Code 88 For cases*).. [ ][ ]

(01) Dental department (04) Out Patient Department (OPD)
(02) Surgery
(03) Medicine

A3. Main Diagnosis of control____

FOR CASES:

A4. Type of cases?.. [ ][ ]

(01) Oral cancer (Go to Q. A7) (02) Oral Leukoplakia.

A5 Oral Leukoplakia Site.. [ ][ ]

(01) Gums	(04) Right Cheek	(07) Middle of the tongue	(10) Floor of the mouth
(02) Lower Lip	(05) Right side of the Tongue	(08) Back of the tongue	(11) Soft Palate
(03) Left Cheek	(06) Left Side of the Tongue	(09) Under surface of the tongue.	
	(12) Oral Leukoplakia with Overlapping Region.		

A6. Extent of the Oral Leukoplakia.. [ ][ ] Mm.

A7. Cancer Site: .... [ ][ ]

(01) Gums	(04) Right Cheek	(07) Middle of the tongue	(10) Floor of the mouth
(02) Lower Lip	(05) Right side of the Tongue	(08) Back of the tongue	(11) Soft Palate
(03) Left Cheek	(06) Left Side of the Tongue	(09) Under surface of the tongue	
(12) Oral Cancer with Overlapping Region			

For All the Subjects:

A8. Date of Diagnosis............................. [ ][ ] Day. [ ][ ] Month [ ][ ][ ][ ] year (99-99-9999 don't know)

A9. Time since Diagnosis (Days).. [ ][ ]

A10. Time since Pre-Diagnostic complaints, sign and symptoms Days/Months............. [ ][ ]

A11. Interviewer's name: ....

A12. Date of Medical Data collection....... [ ][ ] Day [ ][ ] Month [ ][ ][ ][ ] Year

Section B. General Information

B1. Date of interview... ☐☐ Day ☐☐ Month ☐☐☐☐ Year

B2. Time of beginning of the interview............. ☐☐ Hour ☐☐ Minute.

B3. Sex.. ☐☐

(01) Male (02) Female

B4. How old are you? .... ☐☐ Years Old

B5. What is your marital status? .... ☐☐

(01) Single (02) Married (03) Divorced

B6. What is the highest level of education you have completed?... ☐☐

(01) No formal education
(02) Less than primary school completed
(03) Primary school completed
(04) Less than high school completed
(05) High School completed
(06) College, University completed
(07) Postgraduate degree completed
(08) Don't know.
(09) Refused

B7. Which of the following best describes your main work status last 12 months? .... ☐☐

(01) Government Employee
(02) Non-Government employee
(03) Business small
(04) Business Large
(05) Farming
(06) Industrial workers
(07) Daily labourer
(08) Other Self employed
(09) Student
(10) House worker
(11) Retired
(12) Unemployed, able to work
(13) Unemployed, unable to work
(14) Other, Please specify_______

B8. Weight measurement?........................... ☐☐☐ . ☐☐ **Lbs**

B 9. Height Measurement?......................... ☐☐ **Feet** ☐☐ **Inch**

Section C. Smokeless tobacco habits

Interviewer Reminder: Use the life grid if necessary to help answer Q C1 to C4.
- ***Avoid overlapping years for the same product, type of cigarette or amount smoked, i.e. Record 30-40, 41-45 rather than 30-40, 40-45.***
- ***Only note changes occurring in one year or more.***
- ***Exclude quitting during pregnancy(ies) if for less than one year.***

C1. Do/ did you use chewing tobacco, bêtel quid, Areca nut and/or pan masalla etc? ☐☐

(00) Never (Go to C4) (01) Yes, I still do (02) Yes, only in the past

From age	To age (A)	Type (B)	Duration (Minutes)	Consumption (How many)	Per (C)
☐☐	☐☐	☐☐	☐	☐☐	☐☐
☐☐	☐☐	☐☐	☐	☐☐	☐☐
☐☐	☐☐	☐☐	☐	☐☐	☐☐
☐☐	☐☐	☐☐	☐	☐☐	☐☐
☐☐	☐☐	☐☐	☐	☐☐	☐☐
☐☐	☐☐	☐☐	☐	☐☐	☐☐

**To age (A)**	**Type (B)**	**Per (C)**
If still taking smokeless tobacco products, write age of the time of interview	(01) Zarda only<br>(02) Betel quid with Zarda<br>(03) Betel quid without Zarda<br>(04) Areca nut with Zarda<br>(05) Areca nut without Zarda<br>(06) Sadapata only<br>(07) Betel quid with Sadapata<br>(08) Gul<br>(09) Pan masala<br>(10) Betel leaf<br>(11) Other, specify___________	(01) Day<br>(02) Week<br>(03) Month

C2 . When you chew tobacco, where in your mouth you usually keep it, on the left side or the right side? ☐☐
(01) Left (02) Right (03) Both.

C3. Did you ever use snuff?...

(00)No (Go to section D) (01) yes (02) yes, only in the past

From Age	To age (A)	Consumption (How many)	Per (C)

To Age (A)	Per (C)
If still using, write age at the time of interview	(01)Day<br>(02)Week<br>(03)Month

C4. Which brand of snuff you use most often? ______________

C5.When using snuff Do you usually take it by nose or mouth? ....

(01) Nose (Go to next Section) (02) Mouth (03) both.

C6. How long do you keep snuff in your mouth? __________ **Minutes**

C7. When you take snuff, where in your mouth you usually keep it, on the left side or the right side?.......

(01) Left (02) Right (03) Both.

C8. Do you usually place the snuff towards the front or the back of the mouth?...

(01) The front (02) The back (03) The centre.

Section D. Smoking Habits

D1. Have you ever smoked in your life? .... ☐☐

(00) Never (Go to next section) (01) Occasionally (02) Yes (I still do) (03) Yes, but only in the past.

D2. Did you ever Smoke cigarettes? .... ☐☐

(00 No (Go to next Q, D3) (01) Yes (02) Yes, only in the past.

From Age	To age (A)	Type (B)	Consumption (How Many)	Per (C)
☐☐	☐☐	☐☐	☐☐	☐☐
☐☐	☐☐	☐☐	☐☐	☐☐
☐☐	☐☐	☐☐	☐☐	☐☐
☐☐	☐☐	☐☐	☐☐	☐☐
☐☐	☐☐	☐☐	☐☐	☐☐
☐☐	☐☐	☐☐	☐☐	☐☐

To age (A)	Type (B)	Per (C)
If still smoking, write age of the time of interview	(01) Filter<br>(02)Non-Filter<br>(03)Hand-Rolled	(01) Day<br>(02) Week<br>(03) Month

D3. Did you ever smoke bidis? .... ☐☐

(00) No (Go to section.D 4) (01) Yes (02) Yes, only in the past

From Age	To age (A)	Consumption (How many)	Per (C)
☐☐	☐☐	☐☐	☐☐
☐☐	☐☐	☐☐	☐☐
☐☐	☐☐	☐☐	☐☐
☐☐	☐☐	☐☐	☐☐
☐☐	☐☐	☐☐	☐☐

To Age (A)	Per (C)
If still using, write age at the time of interview	(01) Day<br>(02) Week<br>(03) Month

D4. Did you ever smoke, water pipe/ Hukkah? ....

(00) No (Go to Q.D5) (01) Yes (02) Yes, only in the past

From Age **To age (A)** **Consumption (How many pipes full of tobacco)** **Per (C)**

To Age (A)	Per (C)
If still using, write age at the time of interview	(01) Day<br>(02) Week<br>(03) Month

D5. Did/Do you smoke cigar? ....

(00) No (Go to next section) (01) Yes (02) Yes, only in the past

From Age **To age (A)** **Consumption (How many)** **Per (C)**

To Age (A)	Per (C)
If still using, write age at the time of interview	(01) Day<br>(02) Week<br>(03) Month

Section E. Drinking Habits

E1. Did you ever drink alcoholic beverages at least once a month?...... ☐☐

(00) No (Go to next section) (01) Yes, I do (02) Yes Only in the past

E2. When Do/ did you usually drink alcoholic beverages?.................... ☐☐

(01) With meals (03) Both
(02) Between meals (04) Only at social events.

**E3. Beverages (A)**	**if (A) – (05) Then Specify Other beverages**	**From Age**	**To Age**	**Unit (B)**	**Consumption (How Many)**	**Per (C)**
☐☐	☐☐	☐☐	☐☐	☐☐	☐☐	☐☐
☐☐	☐☐	☐☐	☐☐	☐☐	☐☐	☐☐
☐☐	☐☐	☐☐	☐☐	☐☐	☐☐	☐☐
☐☐	☐☐	☐☐	☐☐	☐☐	☐☐	☐☐
☐☐	☐☐	☐☐	☐☐	☐☐	☐☐	☐☐
☐☐	☐☐	☐☐	☐☐	☐☐	☐☐	☐☐
☐☐	☐☐	☐☐	☐☐	☐☐	☐☐	☐☐
☐☐	☐☐	☐☐	☐☐	☐☐	☐☐	☐☐

Beverages (A)	Unit (B)	Per (C)
(01) Local Toddy<br>(02) Wine<br>(03) Bear<br>(04) Hard liquor (>35) (whiskey, vodka, brandy, rum,gin)<br>(05) Other, Specify.......................................	(01) Small glass (50ml) (1-2oz)<br>(02) Medium glass (100ml) (2-3oz)<br>(03) Big glass (250ml) (1/2 pint)<br>(04) ½ small bottle (330ml) (1beer)<br>(05) Bottle (700-750ml) 921oz)	(01) Day<br>(02) Week<br>(03) Month

Section F. Oral Health

I am going to ask you some questions about your oral health before your diagnosis/ being seen at this clinic and at a different time in your lifetime.

F1. Did you wear complete dentures? .... ☐☐

(00) No (Go to question F4) (02) yes, top only

(01) Yes bottom only (go to question F3) (03) yes, top and bottom.

F2. At what age did you start wearing complete top dentures? Years............................... ☐☐☐

F3. At what age did you start wearing complete bottom dentures? Years......................... ☐☐☐

(Code 888 if F1 = 02)

F4. Did you wear partial dentures? .... ☐☐

(00) No (02) yes, bottom only
(01) Yes, top only (03) yes top and bottom.

F5. What instrument you use to clean your teeth?... ☐☐

(01) Toothbrush (02) Fingers (03) Sticks (04) Others, specify_________________.

F6. How often do you clean your teeth? .... ☐☐

(00) Never (03) Every other day
(01) Less than once a week (04) Once a day
(02) 1-2 times a week (05) twice or more a day.

F7. Did you use any kind of substance to clean your teeth? .... ☐☐

(00) No
(01) Charcoal
(02) Toothpaste
(03) Tooth Powder
(04) Other, specify______________________.

F8. Did your gums bleed, when you clean your teeth? .... ☐☐

(00) No (01) Sometimes (02) Always or almost always.

F9. Have you ever had an ulcer or a cut because of denture or a tooth? .... ☐☐

(00) No (01) Yes.

F10. In the last 20 years how often did you see a dentist?.. ☐☐

(00) Never (03) Every 2-5 Years
(01) Every 6 Months (04) Once every 5 years
(02) Every year (05) Only when I had Pain.

Section G. Family cancer History

G1. Has any member of your biological family ever had cancer?.. ☐☐

(00) No (01) Yes (99) Don't know.

**G2.**	**Relationship (A)**	**status (B)**	**Current/ last age (C)**	**Type of Cancer**	**Age at Diagnosis (D)**
	☐☐	☐☐	☐☐☐	____________	☐☐☐
	☐☐	☐☐	☐☐☐	____________	☐☐☐
	☐☐	☐☐	☐☐☐	____________	☐☐☐
	☐☐	☐☐	☐☐☐	____________	☐☐☐
	☐☐	☐☐	☐☐☐	____________	☐☐☐
	☐☐	☐☐	☐☐☐	____________	☐☐☐

Relationship (A)	Status (B)	Current/ Last age (C)	Age of Diagnosis (D)
(01) Mother<br>(02) Father<br>(03) Sister<br>(04) Brother<br>(05) Daughter<br>(06) Son<br>(07) Grand mother<br>(08) Grand Father<br>(09) Aunty/Uncle	(01) Deceased<br>(02) Alive	(999) Don't know<br>If alive write the current age.<br>If deceased, give age at death	(999) Don't know

.
